# The Application of Spleen-Preserving Splenic Regional Laparoscopic Lymphadenectomy with Spleen Kept In Situ and Laparotomy with Spleen Lifted Out of the Abdomen for Locally Advanced Proximal Gastric Cancer: A Retrospective Study

**DOI:** 10.1155/2019/4283183

**Published:** 2019-10-15

**Authors:** Changrong Que, Shuangming Lin, Yibin Zhu, Dongbo Xu

**Affiliations:** Department of Gastrointestinal Surgery, Longyan First Hospital Affiliated with Fujian Medical University, Longyan, 364000 Fujian Province, China

## Abstract

**Background and Purpose:**

Findings whether laparoscopic lymphadenectomy with spleen kept in situ or laparotomy with spleen lifted out of the abdomen is more effective remain inconclusive. This study is aimed at comparing outcomes of spleen-preserving splenic regional laparoscopic lymphadenectomy with spleen kept in situ versus laparotomy with spleen lifted out of the abdomen for locally advanced proximal gastric cancer.

**Methods:**

Data from patients with locally advanced proximal gastric cancer were collected from January 2011 to January 2014. A total of 246 patients were identified who received D2 radical total gastrectomy together with spleen-preserving splenic regional lymphadenectomy. Of those patients, 87 patients underwent laparoscopic splenic regional lymphadenectomy with spleen kept in situ (LSKS-SRLA) and 159 patients underwent laparotomy with spleen lifted out of the abdomen (LSLA-SRLA). Surgical outcomes and long-term outcomes were compared between the two groups.

**Results:**

The total number of lymph node dissection, intraoperative blood loss volume, intraoperative injury cases, and postoperative complications had no statistically significant difference between the two groups. The number of splenic regional lymph node dissections was 3.90 ± 1.05 per case in the LSLA-SRLA group and 2.89 ± 1.04 in the LSKS-SRLA group. The operation time, length of the incision, and hospital days were shorter in the LSKS-SRLA group. The total recurrence and metastatic rates and 3-year cumulative survival rate had no statistically significant difference between the two groups.

**Conclusions:**

Similar long-term outcomes were achieved in the LSKS-SRLA and LSLA-SRLA groups for locally advanced proximal gastric cancer. However, in the aspects of surgical time, length of incision, and postoperative recovery, the LSKS-SRLA group had obvious advantages.

## 1. Introduction

Throughout the development of gastric cancer treatment, although chemotherapy and targeted drugs continue to be developed to a certain extent, which improves the treatment effects of gastric cancer, the overall survival rate is still not satisfactory. Radical surgery is still the only possible cure for gastric cancer. At present, D2 radical resection of gastric cancer is the standard surgical method for locally advanced gastric cancer. The literature shows that the incidence of proximal gastric cancer has increased in both Eastern and Western countries [1]. Especially in China, advanced gastric cancers account for the majority of all gastric cancers. In the last decade, splenectomy or pancreatosplenectomy was needed to achieve R0 resection for locally advanced proximal gastric cancer in Japan, even without direct invasion. However, now, according to the prospective randomized controlled trial JCOG0110, the incidence of No. 10 station lymph node metastases for proximal gastric cancer that did not invade the greater curvature was as low as 2.4%. The latest 4^th^ edition gastric cancer treatment guidelines issued by the Japan Gastric Cancer Association stipulate that complete dissection of No. 10 station lymph node is thought unnecessary unless the tumor invades the greater curvature line. Therefore, this means dissection of No. 10 station lymph nodes may have a positive effect for those patients whose tumors invade the greater curvature line. The procedure of splenic hilar lymph node dissection with spleen preservation still has clinical benefit. However, for the spleen kept in situ or lifted out of the abdomen, laparotomy or laparoscopic surgery, as well as other aspects, are controversial. Laparoscopic lymphadenectomy with spleen kept in situ or laparotomy with spleen lifted out of the abdomen is inconclusive. This study tried to solve this problem by retrospectively analyzing the clinical data of patients with locally advanced proximal gastric cancer at the Department of Gastrointestinal Anal Surgery, Longyan First Hospital Affiliated with Fujian Medical University, China. As far as we know, this is the first report on this topic.

## 2. Methods

### 2.1. Patient Selection

Clinical data of patients with locally advanced proximal stomach cancer was collected by retrieving medical records from January 2011 to January 2014. A total of 246 patients were identified who received D2 radical total gastrectomy together with spleen preservation surgery. Of these patients, 87 patients underwent laparoscopic splenic regional lymphadenectomy with spleen kept in situ (LSKS-SRLA) and 159 patients underwent laparotomy with spleen lifted out of the abdomen (LSLA-SRLA). The inclusion criteria were defined as follows: histologically proven gastric carcinoma, no distant metastases, advanced tumors located in the proximal stomach, gastric cancer patients who received a D2 radical total gastrectomy and an R0 resection with spleen-preserving splenic regional lymphadenectomy, age less than 70 years old, and no major organ dysfunction. The exclusion criteria were defined as follows: stage T4b or distant metastases, treatment with preoperative chemotherapy or radiotherapy, a lack of a pathological diagnosis, remnant gastric cancer, an emergency operation with bleeding or perforation, age more than 70 years old, and with major organ dysfunction. Lymphadenectomy was performed according to the Japanese gastric cancer treatment guidelines. Tumor staging was based on the 7th edition of the pathological (pTNM) classification of the American Joint Committee on Cancer (AJCC). The study was approved by the ethics committee of the Longyan First Hospital Affiliated with Fujian Medical University.

### 2.2. Operative Procedure and Chemotherapy Regimen Selection

Currently, laparoscopic radical total gastrectomy is mainly used for early gastric cancer in China. All patients were informed of the possible complications and given a full detailed explanation of each surgical procedure as well as the advantages and disadvantages of LSKS-SRLA versus LSLA-SRLA. Based on this, all patients selected operative procedure LSKS-SRLA or LSLA-SRLA voluntarily, and written informed consent was obtained prior to the surgery. The surgeon performed the operation according to the patient's preoperative choice. According to postoperative pathological conditions, including tumor pathological type, depth of invasion, lymph node metastasis, and neurovascular invasion, adjuvant chemotherapy was performed using 5-fluorouracil- (5-FU-) based regimens (mostly oxaliplatin with either Xeloda or S1). When recurrence or metastases were found, chemotherapy regimens were changed.

### 2.3. Quality Control of Surgery and Operative Technique

In both groups, D2 radical total gastrectomy was performed by the experienced surgeon Doctor Xu and the same assistants who performed D2 radical total gastrectomy for more than one thousand patients. D2 radical total gastrectomy was carried out according to the 14th edition of the Japanese Gastric Cancer Treatment Protocol. The anastomosis route of the esophagus and jejunum was Roux-en-Y. On this basis, the following two procedures were performed.

**Figure 1:**
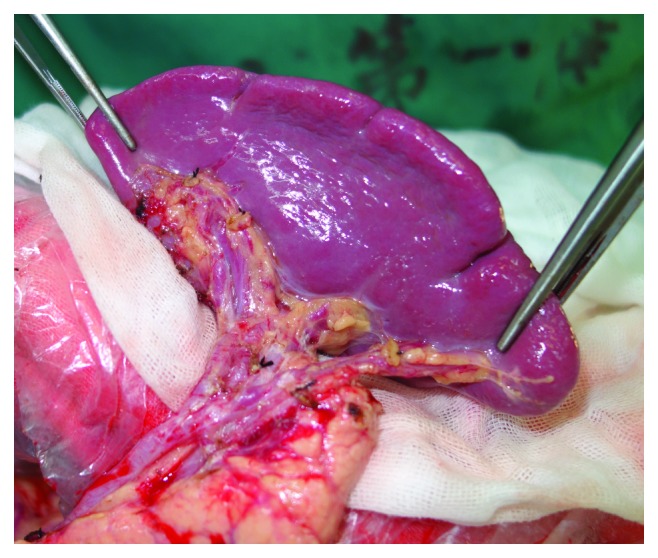
State of splenic region of the LSLA-SRLA group after operation.

*Group LSLA-SRLA:* first, the body and tail of the pancreas were mobilized from the retroperitoneum. Ligaments around the spleen and gastroesophageal junction were cut off. The stomach, spleen, and pancreas were left out of the abdomen. Then, the left gastroepiploic artery was ligated and cut at the origin; the gastrosplenic ligament was severed close to the splenic hilum. No. 4sb and 4sa station lymph nodes were cleaned. Lymphatic adipose tissue around the splenic artery (No. 11p and 11d station) and its branch (No. 10 station) was resected. Lymph node dissection was first performed on the anterior part of the spleen and then the posterior part. After the above steps were completed, the spleen was replaced. The state of the splenic region after the surgery is shown in Figure 1.

**Figure 2:**
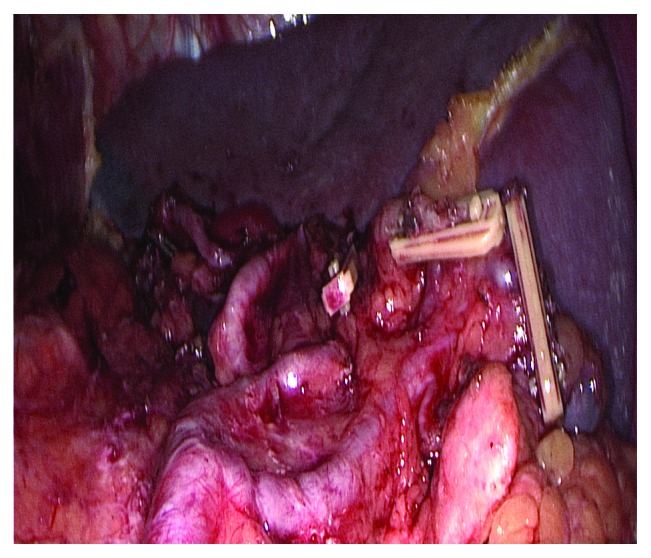
State of splenic region of the LSKS-SRLA group after operation.

*Group LSKS-SRLA:* the pancreas and the spleen were not mobilized. The left gastroepiploic artery was ligated and cut at the origin; the gastrosplenic ligament was severed close to the splenic hilum, and No. 4sb and 4sa station lymph nodes were cleaned. No. 11p and 11d station lymph nodes were resected without pancreatic mobilization. No. 10 station lymph nodes at the splenic artery branch surface were dissected. The state of the splenic region after the surgery is shown in Figure 2.

### 2.4. Follow-Up

All patients were followed up after surgery. Follow-up methods included telephone calls, outpatient records, and hospital examination. Follow-up was performed every 3 months until 3 years after the surgery. All the remaining 237 patients were followed up.

### 2.5. Statistical Analysis

All of the data analysis were performed with the SPSS for windows, version 17.0 (SPSS Inc., Chicago, IL). Categorical variables were analyzed using the Chi-squared test or Fisher's exact test, whereas continuous variables were analyzed using the unpaired Student's *t*-test. Cumulative survival rates were estimated using the Kaplan-Meier method and compared with the log-rank test. Two-sided *P* values less than 0.05 were considered to be significant.

## 3. Results

### 3.1. Patient Characteristics

The clinical characteristics of patients enrolled in the study are shown in Table 1. According to the inclusion criteria, a total of 246 patients who received D2 radical total gastrectomy together with spleen-preserving splenic regional lymphadenectomy were included in our study. Of those patients, 87 patients underwent LSKS-SRLA and 159 patients underwent LSLA-SRLA. Of all patients, more than half of them were men (53.2%), and 115 patients were female. The median age of patients was 54 years (range, 32 to 70 years). There were no significant differences in age, gender, body mass index, and pathological characteristics of the tumor between the LSKS-SRLA and LSLA-SRLA groups.

### 3.2. Surgical Results

Baseline surgical aspects of the included patients per group are shown in Table 2. The comparison of intraoperative injury and postoperative complications are shown in Table 3. Postoperative complications were graded using the Clavien-Dindo system. The total number of lymph node dissection, intraoperative blood loss volume, intraoperative injury cases, and postoperative complications had no statistically significant difference between the two groups. The number of splenic regional lymph node dissections was 3.90 ± 1.05 per case in the LSLA-SRLA group and 2.89 ± 1.04 in the LSKS-SRLA group. The operation time, length of the incision, and hospital days were shorter in the LSKS-SRLA group. There were 3 patients who died of anastomotic leakage, 1 case of pulmonary infection, and 2 cases of myocardial infarction after the surgery in the LSLA-SRLA group and 1 case of anastomotic leakage, 1 case of pulmonary infection, and 1 case of myocardial infarction in the LSKS-SRLA group.

### 3.3. Oncologic Outcomes

**Figure 3:**
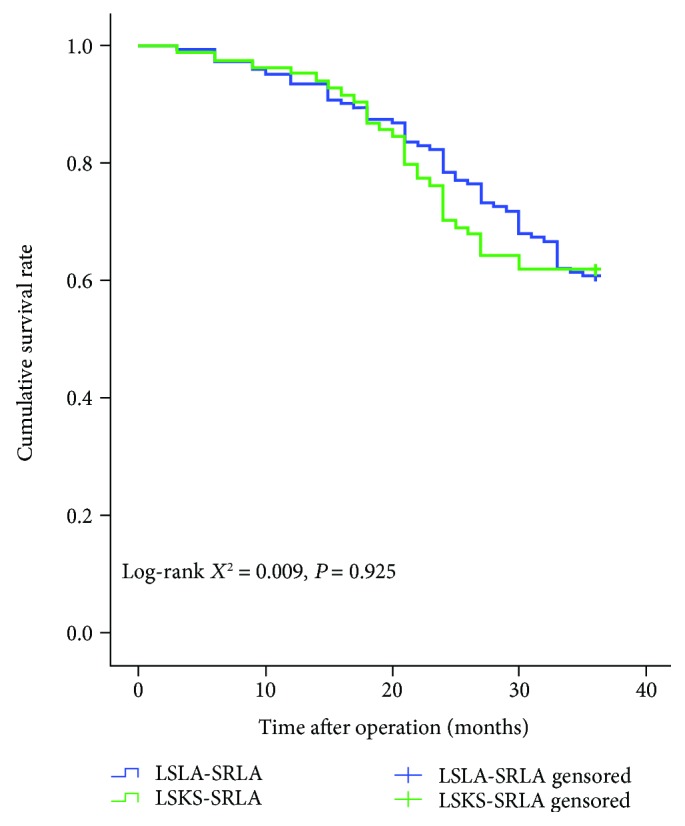
Overall survival of the LSKS-SRLA and LSLA-SRLA groups. The two groups showed a similar result. The follow-up period was 36 months after surgery in each group.

In the LSLA-SRLA group, 64 cases of recurrence and metastases were found. The primary sites of recurrence and metastases were celiac lymph nodes (24 cases), peritoneal (15 cases), liver (11 cases), pulmonary (6 cases), bone (4 cases), and others (4 cases). Fifty-four patients died from the recurrence or metastases, and 6 patients died from other diseases. The total 3-year recurrence and metastatic rate was 41.8%, and the overall survival rate was 60.8%. In contrast to the LSLA-SRLA group, there were 36 patients who suffered from recurrence and metastases in the LSKS-SRLA group. The primary sites of recurrence and metastases were celiac lymph nodes (14 cases), peritoneal (10 cases), liver (6 cases), pulmonary (3 cases), bone (1 case), and others (2 cases). Twenty-nine patients died of recurrent and metastatic disease, and 3 patients died of other diseases. The total 3-year recurrence and metastatic rate was 42.9%, and the overall survival rate was 61.9%. There were no significant differences in the total recurrence and metastatic rates between the two groups (*P* = 0.878), and no significant differences in the survival curve (*P* = 0.925), as shown in Figure 3.

## 4. Discussion

As reported in the literature, the positive rate of splenic regional lymph nodes in proximal gastric cancer was 8.8% to 27.9% [2, 3]. This study showed that the rate was 14.2%. Shin and others reported that the 5-year survival rate of lymph node metastases in the splenic area was significantly reduced [4–6]. Zhu and others found that splenic area lymph node metastases were an independent risk factor for prognosis [2]. The latest 4th JGCA guidelines mention that No. 10 station lymph nodes should be dissected for proximal gastric cancer which invades the greater curvature line, although it is unnecessary for all proximal gastric cancer patients [7]. In China, dissection of No. 10 station lymph nodes was carried out in many medical centers due to the more advanced tumor stage than those seen in Japan. Because of the special anatomical location of the spleen, the traditional surgical exposure provides poor vision. The spleen texture is brittle and its vascular classification is complex. Based on the above reasons, the lymph node dissection of the splenic region has always been a difficult step in gastric cancer operations. Therefore, in order to achieve lymph node dissection, combined splenectomy was often adopted in the last few decades [8]. However, compared with spleen-preserving lymphadenectomy, many studies have shown that splenectomy has a significant increase in surgical complications [7, 9, 10], the survival rate has not improved, and the spleen has important immune functions. Therefore, in recent years, spleen-preserving splenic regional lymphadenectomy has been gradually applied. The literature shows that the spleen-preserving splenic regional lymphadenectomy performed by an experienced gastric cancer specialist did not increase the incidence of associated complications [10, 11].

Without dissociating the spleen, it is very difficult to perform planar anatomy and thorough lymph node dissection from the splenic hilar. LSLA-SRLA can excellently solve the problem of surgical vision exposure. After dissociating the spleen and the body and tail of the pancreas, they were lifted out of the abdominal cavity for lymph node dissection and then were replaced into the abdominal cavity when dissection was over. All operations were performed under direct vision. Therefore, the anatomical structure was clearer. The lymphatic adipose tissue behind the spleen hilar could also be resected. The degree of vascularization was higher, and the bleeding from splenic vessels and its branches could be easily controlled. However, the scope of surgery was complex, and the trauma was obvious, which was an adverse aspect. The operation time was 239.5 ± 35.5 min, which was significantly longer than the LSKS-SRLA group. The total number of lymph node dissection was 46.2 ± 6.3 per case, and the intraoperative blood loss was 183.0 ± 84.7 ml. There was no significant difference compared with the LSKS-SRLA group. In addition, for obese and barrel-chested patients, dissociating the pancreatic body and spleen was a very difficult step, making it easy to damage the adrenal, pancreatic fistula, or colon and causing other complications. Thus, surgeons must have a good understanding of the anatomy and surgical space. What they should be aware of was that the dissociation of the spleen and the complete vascularization of the blood vessels, in theory, increased the risk of spleen torsion and postoperative abdominal hemorrhage. There were 16 cases of intraoperative injury, 1 case of spleen torsion, and 2 cases of abdominal hemorrhage.

Since the trauma was so great in the LSLA-SRLA group, surgeons in the area of gastric cancer have been looking for surgical methods which can achieve good lymph node dissection results with the spleen kept in situ. With the deepening of gastric cancer research and the continuous development of laparoscopic instruments, LSKS-SRLA, which did not require mobilization of the pancreatic body and tail and maintaining the spleen in the original position, has been gradually carried out. Hyung and others reported this for the first time in 2008 [12]. The average number of lymph node dissections in the splenic hilar was 2.7 per case. Laparoscopy solved the problem of narrow surgical field when the spleen was kept in situ, especially in patients with obesity and barrel chest. The amplification could also clearly identify the fascial space and blood vessels and their branches and ensure efficient and accurate completion of splenic regional lymph node dissection. However, it requires the surgeon to have rich experience in gastric cancer surgery, skilled laparoscopic technique, and adept cooperation of the assistant [13]. In recent years, the exploration and optimization of surgical approaches, including the left approach, the middle approach, and the posterior pancreatic approach [14], have been continued in China. Among them, Li et al.'s three-step left approach optimized the surgical procedure and shortened the operation time [15]. It made it possible to promote this difficult technique. Compared with LSLA-SRLA, LSKS-SRLA had the significant advantages of small incision, less trauma, shorter surgical time, and faster postoperative recovery. The incision length was 9.1 ± 1.6 cm, and the operation time was 196.8 ± 12.7 min. The average days of hospitalization were 11.03 ± 2.51 days. However, due to the difficulty in dissection of the rear lymph nodes of the spleen hilar, the average number of lymph nodes removed in the spleen hilar was relatively fewer. The average number was 2.89 ± 1.04 per case, which was significantly different from the LSLA-SRLA group. At present, most scholars believe that the number of lymph nodes behind the spleen is few and the positive rate is very low. This study also confirmed this view indirectly. Although the difference in the number of lymph node dissections in the spleen hilar between the two groups is statistically significant, the number is approximately one, and there was no significant difference between the 3-year overall survival rate and the recurrence and metastatic rates in the two groups, which also confirmed that dissection of the rear lymph nodes of the spleen hilar was of little significance.

What needs to be noted is that the purpose of this study was not to confirm whether the splenic hilar lymph nodes should be dissected. At present, in China, a large-scale multicenter prospective clinical trial CLASS-04 is also underway to verify the efficacy of laparoscopic splenic regional lymphadenectomy with spleen kept in situ. With the development of therapeutic concepts, surgical anatomy techniques, surgical instruments, and lymphatic metastasis mechanism [16], we believe that more and more surgeons in the area of gastric cancer will try to perform laparoscopic splenic regional lymphadenectomy with spleen kept in situ. However, LSKS-SRLA showed lower results between 20 months and 36 months in the survival graph. It needs longer follow-up to explain this phenomenon. Additional randomized controlled clinical trials should be conducted to provide valuable evidence of the safety and efficacy of this surgical procedure.

In conclusion, LSKS-SRLA in proximal locally advanced gastric cancer has the advantages of good exposure, less trauma, and rapid recovery, especially for obese patients and barrel-chested patients. Simultaneously, intraoperative and postoperative complications, recurrence and metastatic rates, and survival rates are not significantly different from the LSLA-SRLA group.

## Figures and Tables

**Table 1:** Comparison of clinicopathological features of the LSKS-SRLA and LSLA-SRLA groups.

Characteristics	LSKS-SRLA (*n* = 87)	LSLA-SRLA (*n* = 159)	*P*
Age	55.02 ± 10.44	53.99 ± 12.16	0.503
Gender			0.212
Male	51	80	
Female	36	79	
BMI (kg/m^2^)	26.39 ± 3.89	26.03 ± 4.04	0.501
pT stage			0.981
T2	8	14	
T3	14	27	
T4a	65	118	
pN stage			0.745
N0	18	28	
N1	20	46	
N2	32	58	
N3	17	27	
pTNM stage			0.868
IB	3	7	
IIA	8	13	
IIB	14	20	
IIIA	19	43	
IIIB	29	56	
IIIC	14	20	
Nerve invasion			0.256
Yes	11	29	
No	76	130	
Lymphovascular invasion			0.435
Yes	16	36	
No	71	123	
Type of tumor differentiation			0.136
High	34	48	
Moderate	39	69	
Low	14	42	

LSKS-SRLA = laparoscopic splenic regional lymphadenectomy with spleen kept in situ; LSLA-SRLA = laparotomy with spleen lifted out of the abdomen.

**Table 2:** Baseline surgical aspects of the included patients per group.

Variable	LSKS-SRLA (*n* = 87)	LSLA-SRLA (*n* = 159)	*P*
Operation time (min)	196.8 ± 12.7	239.5 ± 35.5	0.000
Estimated blood loss (ml)	169.5 ± 83.4	183.0 ± 84.7	0.231
Length of incision (cm)	9.1 ± 1.6	22.2 ± 2.8	0.000
Total no. of retrieved lymph nodes	45.8 ± 5.6	46.2 ± 6.3	0.640
No. of splenic region lymph nodes	2.89 ± 1.04	3.90 ± 1.05	0.000
Postoperative hospital stay (days)	11.03 ± 2.51	14.04 ± 4.03	0.000

LSKS-SRLA = laparoscopic splenic regional lymphadenectomy with spleen kept in situ; LSLA-SRLA = laparotomy with spleen lifted out of the abdomen.

**Table 3:** Inoperative injuries and postoperative complications of the LSKS-SRLA and LSLA-SRLA groups.

Variable	LSKS-SRLA	LSLA-SRLA	*P*
Total inoperative injury	6	16	0.405
Splenic vascular injury	2	4	
Spleen injury	1	3	
Adrenal injury	0	2	
Colon injury	0	3	
Pancreatic injury	3	4	
Total postoperative complications	15	43	0.320
I–II complication	10	28	
III–IV complication	2	9	
V (death)	3	6	
Anastomotic leakage	2	5	
Pancreatic fistula	1	4	
Abdominal infection	1	4	
Intra-abdominal bleeding	1	2	
Lymphatic leakage	3	5	
Spleen torsion	0	1	
Spleen infarction	1	2	
Intestinal obstruction	1	6	
Incision infection	1	5	
Pulmonary infection	3	7	
Myocardial infarction	1	0	

LSKS-SRLA = laparoscopic splenic regional lymphadenectomy with spleen kept in situ; LSLA-SRLA = laparotomy with spleen lifted out of the abdomen.

## Data Availability

The data used to support the findings of this study are available from the corresponding author upon request.
